# Comparative performance of large language models in emotional safety classification across sizes and tasks

**DOI:** 10.3389/frai.2025.1706090

**Published:** 2025-11-27

**Authors:** Edoardo Pinzuti, Oliver Tüscher, André Ferreira Castro

**Affiliations:** 1 Leibniz Institute for Resilience Research, Mainz, Germany; 2 Department of Psychiatry, Psychotherapy and Psychosomatic Medicine, University Medical Center Halle, Halle, Germany; 3 German Center for Mental Health (DZPG), Site Halle-Jena-Magdeburg, Halle, Germany; 4 Department of Psychiatry and Psychotherapy, University Medical Center of the Johannes Gutenberg-University Mainz, Mainz, Germany; 5 School of Life Sciences, Technical University of Munich, Freising, Germany

**Keywords:** large language model (LLM), scaling and fine-tuning, privacy-preserving AI, emotional safety classification, affective computing

## Abstract

Understanding how large language models (LLMs) process emotionally sensitive content is critical for building safe and reliable systems, particularly in mental health contexts. We compare the performance of LLMs of different sizes on two key tasks: trinary classification of emotional safety (safe vs. unsafe vs. borderline) and multi-label classification using a six-category safety risk taxonomy. To support this, we construct a novel dataset by merging several human-authored mental health datasets (> 15K samples) and augmenting them with emotion re-interpretation prompts generated via ChatGPT. We evaluate four LLaMA models (1B, 3B, 8B, 70B) across zero-shot and few-shot settings. Our results show that larger LLMs achieve stronger average performance, particularly in nuanced multi-label classification and in zero-shot settings. However, lightweight fine-tuning allowed the 1B model to achieve performance comparable to larger models and BERT in several high-data categories, while requiring < 2GB VRAM at inference. These findings suggest that smaller, on-device models can serve as viable, privacy-preserving alternatives for sensitive applications, offering the ability to interpret emotional context and maintain safe conversational boundaries. This work highlights key implications for therapeutic LLM applications and the scalable alignment of safety-critical systems.

## 1 Introduction

Large Language Models (LLMs) are increasingly embedded in mental health applications, conversational agents, and therapeutic tools (Bucci et al., 2019; Mitsea et al., 2023; Dehbozorgi et al., 2025; Miner et al., 2017; Yang K. et al., 2024; Lai et al., 2023). This trend raises urgent questions about the emotional safety of these systems, especially when deployed in contexts involving vulnerable populations. An essential prerequisite for responsible deployment is the ability of these models to recognize and regulate emotionally harmful content–ranging from subtle expressions of distress to overtly toxic or dangerous language (Inan et al., 2023).

One of the core challenges in emotionally safe AI lies in the dual effect of model scaling. Larger LLMs are not only more fluent and context-aware but also more capable of generating harmful outputs, owing to their greater exposure to toxic, biased, or stereotyped content in training data (Machlovi et al., 2025; Ganguli et al., 2022; Vidgen et al., 2023; Tosato et al., 2025). In response to these risks, systems like OpenAI's Moderation API (OpenAI, 2023) and LlamaGuard (Inan et al., 2023) have emerged as mitigation strategies, applying taxonomic filters or post hoc classifiers to flag unsafe outputs. While these tools can be effective in general-purpose settings, they largely treat emotional safety as an external moderation problem rather than as a core capability embedded within the model itself.

In parallel, a growing body of work has begun to treat emotional safety as an intrinsic model property–evaluating LLMs directly on mental health datasets and comparing their performance against robust baselines such as BERT. These studies benchmark LLMs on clinically relevant text classification tasks and explore interpretability in psychological domains (Yang K. et al., 2024; Lai et al., 2023; Pinzuti et al., 2025). BERT, in particular, remains a widely adopted reference point in this space, given its strong performance, efficient architecture, and demonstrated ability to approach human-level accuracy on emotionally sensitive classification tasks (Devlin et al., 2018; Lee et al., 2020). While these approaches offer valuable insights, its comparisons involve models with varying architectures and training data, making it hard to isolate the impact of scale or fine-tuning (Zhang et al., 2024). This challenge echoes findings from domain-specific transfer-learning research, where it was shown that targeted fine-tuning of pretrained models can substantially improve performance in specialized contexts such as financial sentiment analysis Ergun and Sefer (2025). Crucially, this prior work collectively overlooks whether small, on-device models–where only 4-16 GB of RAM are typically available for all computations–can, with fine-tuning, achieve safety performance comparable to larger models, a key concern in privacy-sensitive domains such as mental health (Yao et al., 2024). Specifically, these raises two questions: (1) To what extent does scale improve a model's ability to detect or avoid emotionally unsafe content? (2) And can smaller models, given targeted supervision, recover the performance advantages typically attributed to scale?

Here, to address these questions, we investigate the scaling behavior of LLMs in the context of emotional safety classification, with a focus on how model size influences performance under different levels of supervision. Our study targets two core tasks: trinary classification of text contenct safety (safe vs. unsafe vs. borderline) and multi-label classification grounded in Llama guard's six-category emotional risk taxonomy (Inan et al., 2023). To support this analysis, we construct a unified benchmark by merging multiple real-world mental health datasets and augmenting them with reappraisal-style prompts generated by ChatGPT (OpenAI, 2023). We evaluate LLaMA models (Dubey et al., 2024; Touvron et al., 2023) across four scales (1B, 3B, 8B, 70B) under zero-shot, few-shot, and fine-tuned conditions, using BERT for (Reimers and Gurevych, 2019) task-specific baselines. By curating a controlled evaluation framework and conducting a systematic comparison across architectures and training regimes, we identified a performance threshold at the 1B scale: at this point, fine-tuned models begin to approximate the safety capabilities of 70B-parameter LLMs and reach performance levels comparable to strong BERT-based baselines. These findings have critical implications for building deployable, privacy-preserving AI systems that are both emotionally aware and aligned for use in mental health and therapeutic contexts (Calvo and D'Mello, 2010; Gabriel, 2020; Picard, 2000).

## 2 Methods

### 2.1 Dataset construction for three-way and multi-class safety classification

We constructed a novel dataset for emotional safety classification by sourcing original text posts from publicly available online mental health-related datasets, including Dreaddit (DREAD; Turcan and McKeown, 2019), Depression Reddit (DR; Pirina and Çöltekin, 2018), Stress cause detection (SAD; Mauriello et al., 2021), Interpersonal risk factor detector (IRF; Garg et al., 2023), Wellness detection dimension (WD; Sathvik and Garg, 2023) (see Table 1 for sample sizes). These datasets contain user-authored content discussing psychological topics, emotional distress, and other mental health-relevant concerns. For the DREAD and DR datasets, we included only posts classified as stressed or depressed.

**Table 1:** Dataset composition and augmentation statistics.

**Dataset**	**Safe**	**Unsafe**	**Borderline**	**LLaMA guard taxonomy (unsafe only)**					
				**Violence & hate**	**Sexual content**	**Guns & illegal weapons**	**Regulated substances**	**Suicide & self-harm**	**Criminal planning**
DREAD; Turcan and McKeown (2019)	1,702	1,702	1,702	384	38	21	427	768	3
DR; Pirina and Çöltekin (2018)	1,108	1,108	1,108	74	5	7	260	737	0
SAD; Mauriello et al. (2021)	6,215	6,215	6,215	1,655	9	25	1,672	2,073	25
IRF; Garg et al. (2023)	1,918	1,918	1,918	70	5	12	362	1,446	1
WD; Sathvik and Garg (2023)	4,926	4,926	4,926	256	8	40	1249	3,307	1
Total	15,869	15,869	15,869	2,440	65	105	3,970	8,331	30

Columns 2-4 show counts for safe, unsafe, and borderline variants generated per dataset. Columns 5–10 report distribution of unsafe samples across the six LLaMA Guard taxonomy categories (Inan et al., 2023).

Each original post was processed using GPT-3.5 to generate three distinct textual variants. The first variant, labeled **Safe** (session_consistent), retained the emotional context of the original post but was reframed in a positive, supportive, or hopeful manner. The second variant, labeled **Borderline** (borderline), expressed emotional vulnerability, despair, or distress without including explicit harmful acts or risk content. This class often used metaphorical or ambiguous language but avoided any explicit harm categories. The third variant, labeled **Unsafe** (emotionally_risky), contained explicit visual or situational elements tied to one of six LLaMA Guard-style harm categories: violence and hate, sexual content, guns and illegal weapons, regulated or controlled substances, suicide and self-harm, and criminal planning (Inan et al., 2023).

To ensure balance across classes in the initial three-way classification task, each original post was augmented with exactly one safe and one unsafe variant, along with an additional borderline example for extended experiments. For unsafe instances, ChatGPT autonomously selected the specific category (among six predefined risk types) under which to generate the new variant. Detailed sample distributions across categories are provided in Table 1), and the prompt templates used for generation are available in the Supplementary material S1.

To complement automated label generation and assess the ecological validity of the dataset, we conducted a small-scale human validation study. A random subset of 210 text samples (30 per category) were manually reviewed by three training psychotherapists which independently evaluated the text samples to verify category coherence between the synthetic labels (safe, unsafe → category) and human judgment. Manual inspection achieved mean label agreement of ≈85% with the GPT-generated labels, indicating high semantic consistency between synthetic and human interpretations, and the level of the best LLM performance. A representative example of the analyzed text is included below, and aggregate rater frequencies are visualized as a bar plot (Supplementary Figure S2):

“Me witnessing explosions in a war-torn city, reflecting the chaos and devastation of a potential conflict escalation.” (**Violence & Hate**)

This analysis provides an additional layer of qualitative assurance, demonstrating that the synthetic dataset aligns closely with expert human judgments in both semantic labeling and affective interpretation.

### 2.2 Classification tasks and model selection

We evaluated models on two related safety classification tasks. All experiments used models from the LLaMA 3 family (Dubey et al., 2024), at 1B, 3B, 8B, and 70B parameter scales. We used 4-bit quantized versions to assess suitability for on-device deployment. Furthermore, to ensure architectural consistency and internal validity in our scaling analysis, we restricted experiments to the LLaMA model family. Comparing models within a single lineage minimizes confounding factors introduced by differences in pretraining data, tokenizer design, or attention mechanisms that exist across other architectures. This design choice enables a clearer interpretation of how model size and supervision regime affect safety classification performance, independent of other architectural biases. Such methodological control aligns with established best practices in scaling research and ensures that observed effects reflect scaling behavior rather than cross-architecture variability (Kaplan et al., 2020).

The first was a **trinary classification** task (**Safe**, **Unsafe**, **Borderline**) using the balanced dataset described in Section 2.1 (Table 1). This setup assessed each model's ability to distinguish clearly safe content from clearly unsafe content, while also handling ambiguous borderline cases. We compared LLaMA models under zero-shot prompting and few-shot prompting. Zero-shot prompting uses category names during inference without training, while few-shot prompting extends this by including 2–4 in-context examples per category, without updating model weights. (see Supplementary material S1 for prompting code).

The second task was a **multi-class taxonomy classification**, implemented as a two-stage pipeline. In Stage **A**, models performed binary classification (**Safe** vs. **Unsafe**; borderline cases excluded). In Stage **B**, unsafe posts were assigned to one of six LLaMA Guard–style harm categories (Inan et al., 2023). We tested each LLaMA model under three supervision regimes: zero-shot prompting, few-shot prompting, and lightweight fine-tuning using LoRA adapters (Hu et al., 2022). For comparison, as a supervised reference model, we included DistilBERT (Sanh et al., 2019), a distilled version of BERT (Devlin et al., 2018) that reduces model size while retaining 97% of its language understanding performance. We used the distilbert-base-uncased implementation from the Hugging Face Transformers library. The model was fine-tuned for 3 epochs on a training portion of our dataset and evaluated on a held-out test set to ensure comparability with LLaMA models. Default hyperparameters were used.

To address class imbalance in Stage **B**, we adopted two evaluation setups. The first was a full taxonomy evaluation, following the LLaMA Guard protocol (Inan et al., 2023), in which categories were evenly subsampled to ensure representation across all six harm types. Because several categories contained fewer than 30 available posts, this setup was evaluated as a single run, and no standard deviations are reported. Running multiple seeds under these conditions would have repeatedly drawn from the same limited samples, producing artificial variance rather than meaningful replication. The second setup focused on high-data categories–the three harm types with sufficient examples for stable evaluation–where we sampled 100 posts per category and repeated the evaluation over five independent runs with different random seeds to estimate mean and standard deviation. This approach balances broad taxonomy coverage with statistical reliability, providing both comprehensive and robust assessments within the constraints of the available data.

All results are reported in terms of standard classification metrics: **accuracy**, **precision**, **recall**, and the **F1-score**. Accuracy measures the proportion of correctly classified instances:


$$\text{Accuracy}=\frac{\text{TP}+\text{TN}}{N},$$

where TP and TN denote true positives and true negatives, and *N* is the total number of samples.

Precision quantifies the proportion of predicted positive instances that are correct:


$$\text{Precision}=\frac{\text{TP}}{\text{TP}+\text{FP}},$$

where FP represents false positives.

Recall (or sensitivity) measures the proportion of actual positive instances that are correctly identified:


$$\text{Recall}=\frac{\text{TP}}{\text{TP}+\text{FN}},$$

where FN denotes false negatives.

The F1-score is the harmonic mean of precision and recall, providing a balanced measure of model performance:


$$\text{F1}=2\times \frac{\text{Precision}\times \text{Recall}}{\text{Precision}+\text{Recall}}.$$

For multi-class or multi-label settings, we report both *macro-averaged* and *weighted-averaged* variants of these metrics, where each class is either weighted equally (macro) or proportionally to its frequency (weighted) in the evaluation dataset.

### 2.3 Comparison against Qwen models

To evaluate the generalizability of our findings beyond the LLaMA architecture, we additionally tested models from the Qwen 2.5 family, including parameter scales of 0.5B, 3B, 7B, and 72B (Team et al., 2024). These models were selected for their comparable scale distribution and open-weight availability. For parity with the LLaMA experiments, we employed 4-bit quantized versions to maintain similar memory efficiency and to assess suitability for local or edge-device deployment. The inclusion of Qwen models served as a cross-architecture benchmark, providing a secondary validation of scaling trends under identical supervision and evaluation settings. Despite architectural and tokenizer differences, the Qwen models exhibited virtually identical performance trajectories across supervision regimes (Supplementary Figure S5, Supplementary Table S2).

### 2.4 Few-shot prompting

To evaluate supervision effects under constrained conditions, we designed a few-shot prompting setup that balances informativeness with the computational limits of small-parameter models. Each prompt consisted of a single *safe* and multiple *unsafe* examples representing the target taxonomy categories, followed by the input instance to be classified. The full template is provided in the Supplementary Figure S1. A randomized ordering of examples was also tested to rule out positional bias, and performance differences were statistically insignificant.

Although the nominal context window for the small models is 2,048 tokens, extending the prompt with additional demonstrations was avoided for both theoretical and empirical reasons. Prior research has shown that small models exhibit rapid degradation in long-context retention, losing attention to early examples even within the formal context limit Liu et al. (2023). Moreover, few-shot generalization effects emerge primarily at larger scales; models below approximately one billion parameters show minimal benefit–and sometimes reduced accuracy–when provided with many in-context examples (Brown et al., 2020). Finally, excessive or stylistically diverse demonstrations can introduce confusion and label copying, especially in compact models that rely on format cues rather than deep semantic abstraction Min et al. (2022).

For these reasons, we adopted a minimal, format-consistent few-shot configuration that preserved prompt clarity, interpretability, and efficiency while remaining within the computational and memory budgets of on-device deployment scenarios. This design ensures that observed differences in performance primarily reflect supervision level and model scale, rather than confounding effects of prompt length or positional degradation.

### 2.5 Fine-tuning strategy for the 1b-parameter model

To evaluate whether smaller-scale models can approximate the emotional safety classification performance of larger LLaMA models, we fine-tuned only the 1B-parameter model. This decision reflects two core objectives: first, to explore whether privacy-preserving models suitable for on-device deployment can perform competitively; and second, to assess whether task-specific supervision can recover performance typically achieved by larger models, without incurring the computational cost of scaling (Zhang et al., 2024).

#### 2.5.1 Model and optimization

We fine-tuned the LLaMA-3.2-1B-Instruct model (Unsloth implementation; Singhapoo et al., 2025) for supervised classification on mental health–related prompts. The model was initialized in 4-bit quantization (bnb-4bit) to reduce GPU memory requirements. We applied parameter-efficient fine-tuning with LoRA adapters on attention and MLP projection layers (q_proj, k_proj, v_proj, o_proj, gate_proj, up_proj, down_proj). LoRA parameters were set to rank *r* = 16 with α = 16 and no dropout. Gradient checkpointing was enabled to support long context windows with reduced memory overhead (Hu et al., 2022).

#### 2.5.2 Training procedure

Prompts were formatted as chat messages in an instruction–response schema, following common practices in supervised fine-tuning of instruction-tuned models (Ouyang et al., 2022; Taori et al., 2023). The model was trained to output structured labels (Safe/Unsafe and, when Unsafe, one of six taxonomy categories). To align training with outputs, we applied the train_on_responses_only transformation, restricting loss computation to assistant responses. Training was performed using Hugging Face's SFTTrainer (von Platen et al., 2023) with the following settings: context length 1024 tokens, effective batch size 8 (batch size 2 with gradient accumulation), optimizer adamw_8bit, learning rate 5 × 10^−5^ with linear decay, weight decay 0.01, and warm-up steps 5. Precision was FP16 on Turing/Volta GPUs and BF16 on Ampere GPUs. Models were trained for one epoch across the dataset.

#### 2.5.3 Evaluation

The fine-tuned model was evaluated on held-out test prompts stratified by taxonomy (*n* = 100 per class, repeated across runs). For each run, we recorded safe/unsafe predictions and taxonomy labels, along with raw responses. Accuracy was computed per taxonomy and averaged across runs to assess stability and category-level performance.

### 2.6 Measuring VRAM usage at inference time

To estimate the memory efficiency of each model, we measured peak GPU memory usage (VRAM) during inference on a representative batch of examples from the multi-label taxonomy classification task. All models were evaluated using the same hardware environment (NVIDIA A100 40GB) with PyTorch's built-in memory tracking utilities. Specifically, we used torchċudaṁax_memory_allocated() to log the maximum memory allocated by each model during forward pass execution. This metric captures the effective VRAM required to run a model in real-time classification scenarios and reflects a practical upper bound for deployment on resource-constrained devices. Quantized (4-bit) versions of LLaMA models were used to simulate realistic low-footprint deployments.

## 3 Results

### 3.1 Few-shot supervision improves multi-label emotional classification, especially for small models

To evaluate whether models can detect specific types of unsafe content, we tested multi-label taxonomy classification using six LLaMA Guard-style harm categories: criminal planning, guns and illegal weapons, regulated substances, sexual content, suicide and self-harm, and violence and hate. This task demands finer-grained safety reasoning beyond binary classification and reflects real-world moderation challenges (Inan et al., 2023; Ganguli et al., 2022).

In the zero-shot (ZS-1) condition, smaller models struggled to recognize most unsafe categories (Table 2). For example, the 1B model showed poor performance across all categories, with mean accuracy ZS-1 1B = 0.000, failing to identify any risk types. The 3B and 8B models demonstrated moderate improvements (mean accuracy ZS-1 3B = 0.290, 8B = 0.432), especially in categories like suicide and violence. The 70B model achieved the best overall results in this setting (mean accuracy ZS-1 70B = 0.582), though its performance remained uneven across categories, with certain risk types still under-recognized.

**Table 2:** Trinary classification results (safe vs. unsafe vs. borderline).

**Model**	**Setting**	**Accuracy ± Std**
Llama-3.2-1B-Instruct	Zero-shot	0.495 ± 0.013
Llama-3.2-3B-Instruct	Zero-shot	0.509 ± 0.010
Llama-3.1-8B-Instruct	Zero-shot	0.487 ± 0.009
Llama-3.3-70B-Instruct	Zero-shot	0.661 ± 0.012
Llama-3.2-1B-Instruct	Few-shot	0.803 ± 0.024
Llama-3.2-3B-Instruct	Few-shot	0.780 ± 0.009
Llama-3.1-8B-Instruct	Few-shot	0.806 ± 0.017
Llama-3.3-70B-Instruct	Few-shot	0.874 ± 0.018

Accuracy (± standard deviation) of LLaMA models (1B, 3B, 8B, 70B) under zero-shot and few-shot prompting. Each condition used three labeled examples per class in the few-shot setting.

Providing just labeled examples per category in the few-shot (FS-1) condition dramatically boosted classification performance for smaller models (see Supplementary material S1, Table 2). The 1B model was able to identify several unsafe categories (mean accuracy FS-1 1B = 0.176), including perfect performance in suicide detection. The 3B and 8B models also benefited significantly (mean accuracy FS-1 3B = 0.486, 8B = 0.672), narrowing the gap with the 70B model, which reached FS-1 mean accuracy = 0.735. Overall, few-shot prompting (FS-1) led to consistent gains across all model sizes. The average improvement across all models from ZS-1 to FS-1 was approximately 39.4%, highlighting the power of minimal supervision to unlock latent safety capabilities even in lightweight models.

Despite these improvements, many categories exhibit non-monotonic scaling in both zero-shot and few-shot settings. This may reflect sensitivity to prompt phrasing, variance introduced by single-run evaluation. As in prior scaling literature, larger models may become more sensitive to subtle or borderline unsafe cues, leading to trade-offs in precision and recall depending on supervision and context (Tosato et al., 2025; Zhang et al., 2024).

### 3.2 High-data evaluation confirms robustness of scaling trends

To assess the stability of scaling patterns and supervision effects under more reliable conditions, we conducted a high-data evaluation using the three taxonomy categories with the largest sample sizes–suicide and self-harm, violence and hate, and sexual content–each (see Table 3). Unlike earlier evaluations, which were based on a single run with 30 posts per class (ZS-1, FS-1), this analysis employed five independent runs of 100 posts (ZS-5, FS-5), offering a more robust estimate of model behavior under reduced variance and class imbalance.

**Table 3:** Multi-label taxonomy classification results across unsafe categories.

**Category**	**1B**					**3B**				**8B**				**70B**			
	**ZS-1**	**ZS-5**	**FS-1**	**FS-5**	**FT-5**	**ZS-1**	**ZS-5**	**FS-1**	**FS-5**	**ZS-1**	**ZS-5**	**FS-1**	**FS-5**	**ZS-1**	**ZS-5**	**FS-1**	**FS-5**
Criminal planning	0.000	–	0.214	–	–	0.038	–	0.407	–	0.471	–	0.769	–	0.842	–	0.905	–
Guns & illegal weapons	0.000	–	0.067	–	–	0.033	–	0.207	–	0.138	–	0.200	–	0.467	–	0.367	–
Regulated / controlled substances	0.000	0.000 ± 0.000	0.000	0.000 ± 0.000	**0.78** **±** **0.03**	0.071	0.037 ± 0.018	0.286	0.431 ± 0.428	0.107	0.113 ± 0.032	0.643	0.625 ± 0.265	0.207	0.239 ± 0.046	0.621	0.693 ± 0.043
Sexual content	0.000	–	0.192	–	–	0.269	–	0.222	–	0.500	–	0.481	–	0.333	–	0.652	–
Suicide & self harm	0.000	0.000 ± 0.000	0.556	0.867 ± 0.298	**0.81** **±** **0.03**	1.000	1.000 ± 0.000	0.567	0.481 ± 0.322	1.000	0.998 ± 0.005	0.862	0.899 ± 0.060	1.000	1.000 ± 0.000	0.964	0.968 ±0.018
Violence & hate	0.000	0.000 ± 0.000	0.148	0.262 ± 0.369	**0.99** **±** **0.01**	0.333	0.403 ± 0.034	1.000	0.913 ± 0.087	0.375	0.418 ± 0.162	0.667	0.793 ± 0.073	0.643	0.736 ± 0.059	0.833	0.799 ± 0.043
Mean (all unsafe categories)	0.000	0.000 ± 0.000	0.208	0.376 ± 0.048	**0.86** **±** **0.07**	0.290	0.480 ± 0.012	0.448	0.608 ± 0.169	0.432	0.510 ± 0.060	0.604	0.772 ± 0.091	0.582	0.659 ± 0.023	0.724	0.820 ± 0.017

Each row corresponds to a LlamaGuard-style taxonomy categories: Criminal planning, Guns & illegal weapons, Regulated/controlled substances, Sexual content, Suicide & self-harm, and Violence & hate (Inan et al., 2023). Models tested include LLaMA-3 family -1B, 3B, 8B, and 70B (Dubey et al., 2024). Columns are grouped by model size and supervision type, with color-coded columns indicating the experimental condition: ZS-1 (light purple), zero-shot prompting with 1 run; ZS-5 (cyan), zero-shot with 5 runs; FS-1 (green), few-shot prompting with 1 run; FS-5 (lime), few-shot prompting with 5 runs. FT-5 (orange), supervised fine-tuning with 5 runs, only for the 1B model. The final row reports mean accuracy across all unsafe categories. Standard deviations are provided where multiple runs were conducted. Few-shot prompts were generated with randomized order of category examples to rule out positional bias. Bold values represent results for the fine-tuned models.

Across all models and categories, few-shot performance (FS-5) remained highly consistent with the original single-run results (FS-1; Table 3). This confirms that the original few-shot evaluations captured stable patterns, and that scaling effects persist under more statistically reliable sampling conditions. Moreover, zero-shot prompting (ZS-5) also yielded results that align with prior single-run observations. Models that underperformed in the single-run setting continued to do so (e.g., 1B maintained accuracy = 0.000), while larger models like 3B and 8B showed only modest variation. These findings suggest that earlier non-monotonicities (e.g., 3B outperforming 8B in one category) were not due to instability or noise, but rather reflect real differences in model sensitivity to content types. Consistent with the accuracy-based trends, additional metrics–precision, recall, and F1-scores–showed comparable relative performance across models and supervision regimes (see Supplementary Table S1; see also confusion matrices Supplementary Figures S3, S4).

Taken together, this high-data analysis strengthens the overall conclusions of the study: scaling trends in emotional safety classification are reliable, and few-shot supervision provides robust gains across model sizes, even in categories where zero-shot performance is low. The consistency between FS-1 and FS-5 confirms that earlier results were not artifacts of limited data, and highlights the importance of sample size in safety-critical classification tasks.

### 3.3 Fine-tuning rescues taxonomy performance for the 1B model

To assess whether lightweight fine-tuning can compensate for limited model scale in emotionally sensitive classification, we fine-tuned the LLaMA-1B model using LoRA adapters and compared its accuracy against (i) the best-performing LLaMA models under few-shot supervision (FS-5), and (ii) a strong BERT baseline. BERT is included as a widely used benchmark in safety-sensitive NLP tasks, often matching or exceeding human-level performance in classification under supervised settings Devlin et al. (2018); Lee et al. (2020).

**Figure 1:**
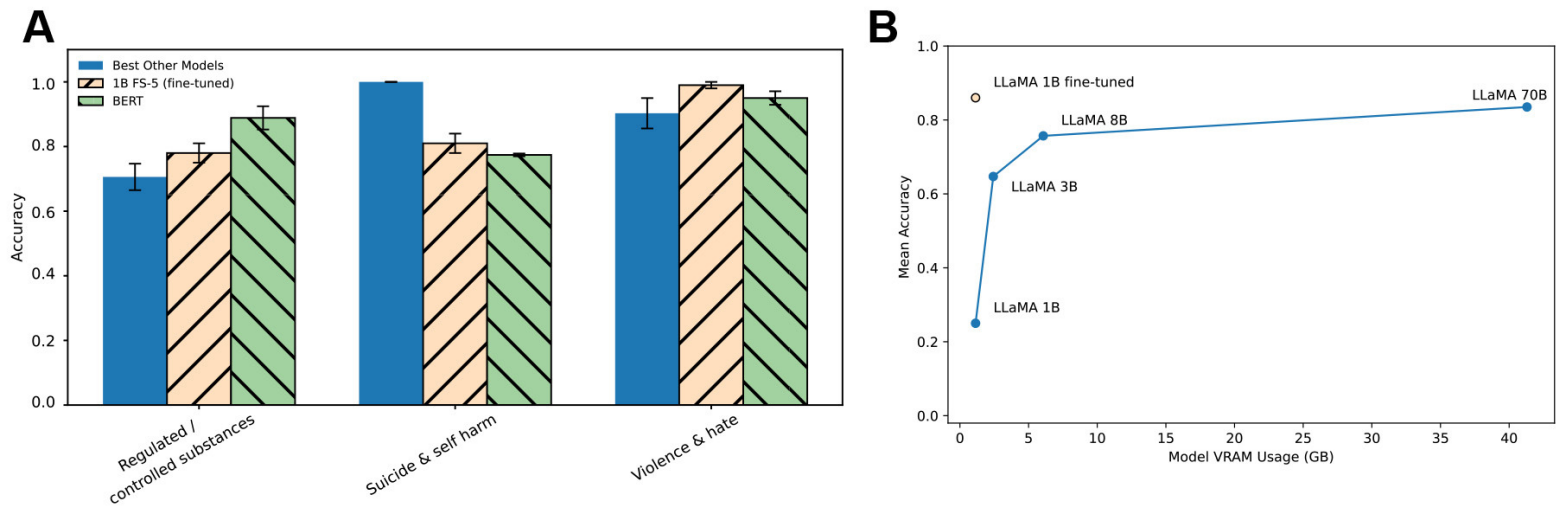
Fine-tuned 1B model performance compared with larger LLaMA models and BERT. **(A)**, Accuracy across three high-data taxonomy categories (Regulated Substances, Suicide & Self-Harm, Violence & Hate). Results are shown for fine-tuned LLaMA-1B (orange striped bars), the best-performing LLaMA models under few-shot prompting (blue solid bars), and a supervised BERT baseline (green striped bars). Error bars show standard deviation across five runs. **(B)**, Mean accuracy across these categories plotted against peak GPU memory (VRAM) usage during inference. Blue line: scaling trend across LLaMA 1B-70B models under few-shot prompting (FS-5). Orange point: fine-tuned 1B model. Lower VRAM usage indicates greater suitability for on-device deployment.

As shown in Figure 1A, the fine-tuned LLaMA-1B model performed competitively across all three high-data categories. On regulated substances, it achieved accuracy = 0.78, outperforming both the strongest LLaMA (70B) model under FS-5 (accuracy = 0.689) and BERT (accuracy = 0.77). On suicide & self-harm, it achieved accuracy = 0.81, falling short of the LLaMA-70B FS-5 (accuracy = 0.950), but comparable to BERT (accuracy = 0.793). On violence & hate, the 1B fine-tuned model reached accuracy = 0.99, exceeding both LLaMA-70B FS-5 (accuracy = 0.849) and BERT (accuracy = 0.955). These results demonstrate that with modest supervision, a small 1B model can match or even exceed the classification accuracy of models that are over 70 times larger, and of strong supervised baselines like BERT.

We further compared mean accuracy across all categories in the FS-5 condition against peak GPU memory requirements (Figure 1B). The fine-tuned 1B model reached mean accuracy = 0.86 using under 2GB of VRAM, outperforming the 3B and 8B models and closely matching the 70B model (mean accuracy = 0.835), while using < 2GB of VRAM–over 20 × less memory than the 70B model. This shows that performance scaling is not strictly tied to parameter count–strategic fine-tuning can dramatically improve efficiency and effectiveness.

In summary, fine-tuning allows a 1B model to reach comparable performance to larger LLaMA models and strong supervised baselines like BERT in high-data safety classification tasks. This supports the feasibility of *privacy-preserving, on-device* deployments, where computational efficiency and user trust are both critical.

## 4 Discussion

### 4.1 Scaling and supervision effects in emotional safety classification

This work set out to investigate how model scale and supervision level affect the ability of large language models (LLMs) to detect emotionally unsafe content. Using a controlled set of LLaMA 3 models ranging from 1B to 70B parameters (Dubey et al., 2024), we examined binary safe, vs. unsafe, vs. borderline classification, multi-label classification across a six-category safety taxonomy, and a high-data subset of three categories (Inan et al., 2023). Our results show that larger LLaMA models generally achieved stronger performance in zero-shot settings, particularly for nuanced multi-label classification where scaling effects were most pronounced. However, few-shot prompting substantially closed the gap between smaller and larger models in the trinary task, showing that even lightweight models can reliably distinguish safe, unsafe, and borderline content with minimal supervision. In contrast, the multi-label taxonomy task remained more sensitive to scale, with larger models showing clear advantages in recognizing fine-grained categories of unsafe content. Strikingly, we found that even the 1B model–despite being 70 times smaller and requiring over 20 × less VRAM–was able to match the performance of the 70B model and a BERT baseline when fine-tuned. This result demonstrates that performance gains attributed to scale can, in part, be recovered through targeted fine-tuning, making compact models a viable option for emotionally sensitive applications.

While the present study focused on the scaling and alignment behavior within the LLaMA model family, future work should extend these analyses to a broader set of architectures to further validate generalizability. Our primary objective was to examine scalable alignment and on-device feasibility under consistent architectural and quantization conditions, rather than to establish inter-model performance rankings. Nevertheless, we conducted an additional comparison using Yang K. et al. (2024) of comparable parameter sizes, which exhibited virtually identical performance trends across supervision regimes (Supplementary Figure S5, Supplementary Table S2). This suggests that the observed scaling relationships are not architecture-specific, although Qwen models differ in quantization and runtime characteristics. Expanding such benchmarks to include additional open-weight models remains a valuable direction for future research, particularly for evaluating trade-offs between safety alignment, efficiency, and deployability.

### 4.2 Implications for safety alignment in mental health applications

Our findings extend ongoing discussions around moderation APIs and taxonomic safety classifiers such as OpenAI's Moderation API (OpenAI, 2023) and LlamaGuard (Inan et al., 2023), which treat safety primarily as an external filtering step. By contrast, our results demonstrate that emotional safety can be embedded as a core capability of the model itself. The observation that even a 1B model, once fine-tuned, reaches parity with both BERT baselines (Devlin et al., 2018; Lee et al., 2020) and the 70B LLaMA highlights a critical threshold: smaller models, given targeted supervision, can recover much of the safety performance often attributed to scale. Whereas prior work has shown that parallelizing model inference across eight edge devices was still required to run full-precision LLaMA-2 70B, our results demonstrate that a fine-tuned 1B model is sufficient for emotional safety classification and can operate entirely on a single edge device within realistic memory limits (Yu et al., 2024).

This has two broader implications. First, it opens the door to lightweight, on-device systems that preserve privacy—an essential condition for deployment in mental health and therapeutic contexts (Yao et al., 2024; Lai et al., 2023). Second, it reframes emotional safety not as a post hoc moderation problem, but as an intrinsic function of human-AI collaboration, enabling systems that can respond adaptively to users affective states rather than merely flagging harmful outputs. This conceptual shift aligns with recent work in affective computing (Picard and Cosier, 1997; Calvo and D'Mello, 2010; Yang K. et al., 2024) and suggests a new generation of cognitive-affective interfaces where safety is seamlessly integrated into dialogue. In this way, our study provides a bridge between scalable alignment research and the design of neuroadaptive or therapeutic tools that can operate reliably under real-world constraints (Friha et al., 2024; Li et al., 2024; Yu et al., 2024).

### 4.3 Limitations of data, task design, and scaling interpretations

While our results are robust across settings, a number of factors should be noted when considering their generalizability. First, our dataset construction—pairing real-world unsafe posts with LLM-generated reappraisals—may introduce stylistic artifacts that models could exploit, potentially inflating performance on the safe vs. unsafe split. Second, the six-category taxonomy is imbalanced, with certain categories underrepresented (Table 1), which may have limited model sensitivity to less frequent harms. Third, our analysis was restricted to the LLaMA family in order to control for architecture, tokenizer, and pretraining corpus (Dubey et al., 2024). While this choice allowed us to isolate the effects of scale and supervision, it also means that our conclusions may not transfer directly to other architectures such which differ in alignment and fine-tuning strategies (Zhang et al., 2024; Tosato et al., 2025). Fourth, although we confirmed scaling trends using higher-data subsets with multiple runs, our full six-category evaluations were based on single runs without multiple seeds, leaving open the possibility of variance effects.

While the six-category taxonomy adopted from LlamaGuard was originally developed for general safety moderation, its structured hierarchy provides a practical and interpretable framework for emotionally relevant risk classification. Our rationale for using this taxonomy was to leverage a well-established, open-source safety schema that has undergone empirical validation for broad harmful-content detection (Dubey et al., 2024). Although certain categories (e.g., “Guns & Illegal Weapons”) are not directly related to emotional risk, others–such as “Self-Harm,” “Hate,” and “Sexual Content”–map closely to constructs of psychological threat and interpersonal distress in digital mental health contexts (Cho and Rader, 2020). Importantly, employing this taxonomy allows for cross-domain comparability with existing safety benchmarks while ensuring that alignment procedures remain reproducible across foundation models. We acknowledge, however, that emotional safety extends beyond content moderation to encompass empathy, tone, and affective intent, which are underrepresented in current taxonomies. Future work should incorporate psychologically grounded taxonomies that integrate emotional valence, empathy markers, and contextual sensitivity (Kirk et al., 2025), bridging the gap between computational safety alignment and clinically informed affective modeling.

Finally, while a full ablation study was beyond the scope of this exploratory comparison, our results nonetheless highlight which methodological components likely contributed most to performance differences across supervision regimes. In particular, we observed that prompt design (zero-shot vs. few-shot) and adapter-based fine-tuning exerted the greatest influence on safety classification accuracy, consistent with recent findings that contextual framing and low-rank adaptation layers substantially affect alignment quality in small models (Hu et al., 2022) (Table 3). From a methodological standpoint, these insights offer an implicit ablation, by isolating key factors that drive model sensitivity and robustness without the need for exhaustive retraining. Future work should formalize these observations through controlled modular ablations, leveraging emerging efficient fine-tuning methods such as AdaLoRA and DoRA (Zhang et al., 2023; Liu et al., 2024), which enable fine-grained parameter updates while maintaining low computational overhead. Such work would further clarify how architectural and supervision components interact to support safe, emotionally aware model behavior at scale.

### 4.4 Conclusion

In sum, this study demonstrates that emotional safety classification in LLMs is not solely a function of scale. While larger models outperform smaller ones in zero-shot and nuanced multi-label settings, few-shot prompting markedly reduces these differences, and fine-tuning allows even a 1B model to have comparable performance of models 70 times larger as well as strong BERT baselines—while using over 20 × less VRAM. These findings highlight that safety can be embedded directly within lightweight models, enabling privacy-preserving and resource-efficient deployment in sensitive domains such as mental health support. More broadly, they suggest that generative AI can be harnessed not only to moderate but also to proactively structure safe and emotionally attuned interactions. Looking forward, integrating such compact, fine-tuned models into affective computing, digital mental health interventions and technologies offers a promising direction for building collaborative systems that are both safe and scalable.

## Data Availability

The raw data supporting the conclusions of this article will be made available by the authors, without undue reservation.

## References

[B1] Brown T. Mann B. Ryder N. Subbiah M. Kaplan J. D. Dhariwal P. . (2020). Language models are few-shot learners. Adv. Neural Inf. Process. Syst. 33, 1877–1901. doi: 10.48550/arXiv.2005.14165

[B2] Bucci S. Schwannauer M. Berry N. (2019). The digital revolution and its impact on mental health care. Psychol. Psychother. 92, 277–297. doi: 10.1111/papt.12222 30924316

[B3] Calvo R. A. D'Mello S. (2010). Affect detection: an interdisciplinary review of models, methods, and their applications. IEEE Trans. Affect. Comp. 1, 18–37. doi: 10.1109/T-AFFC.2010.1

[B4] Cho J. Rader E. (2020). The role of conversational grounding in supporting symbiosis between people and digital assistants. Proc. ACM on Human-Comp. Interact. 4, 1–28. doi: 10.1145/3392838

[B5] Dehbozorgi R. Zangeneh S. Khooshab E. Nia D. H. Hanif H. R. Samian P. . (2025). The application of artificial intelligence in the field of mental health: a systematic review. BMC Psychiatry 25:132. doi: 10.1186/s12888-025-06483-2 39953464 PMC11829440

[B6] Devlin J. Chang M.-W. Lee K. Toutanova K. (2018). Bert: pre-training of deep bidirectional transformers for language understanding. arXiv [preprint] arXiv:1810.04805. doi: 10.48550/arXiv.1810.04805

[B7] Dubey A. Jauhri A. Pandey A. Kadian A. Al-Dahle A. Letman A. . (2024). The llama 3 herd of models. arXiv [preprint] arXiv:2407.21783. doi 10.48550/arXiv.2407.21783.

[B8] Ergun Z. E. Sefer E. (2025). Finsentiment: predicting financial sentiment through transfer learning. Intellig. Syst. Account. Finance Managem. 32, e70015. doi: 10.1002/isaf.70015

[B9] Friha O. Ferrag M. A. Kantarci B. Cakmak B. Ozgun A. Ghoualmi-Zine N. (2024). LLM-based edge intelligence: A comprehensive survey on architectures, applications, security and trustworthiness. IEEE Open J. Commun. Soc. 5, 5799–5856. doi: 10.1109/OJCOMS.2024.3456549

[B10] Gabriel I. (2020). Artificial intelligence, values, and alignment. Minds Mach. 30, 411–437. doi: 10.1007/s11023-020-09539-2

[B11] Ganguli D. Lovitt L. Kernion J. Askell A. Bai Y. Kadavath S. . (2022). Red teaming language models to reduce harms: Methods, scaling behaviors, and lessons learned. arXiv [preprint] arXiv:2209.07858. doi: 10.48550/arXiv.2209.07858

[B12] Garg M. Shahbandegan A. Chadha A. Mago V. (2023). An annotated dataset for explainable interpersonal risk factors of mental disturbance in social media posts. arXiv [preprint] arXiv:2305.18727. doi: 10.18653/v1/2023.findings-acl.757

[B13] Hu E. J. Shen Y. Wallis P. Allen-Zhu Z. Li Y. Wang S. . (2022). Lora: Low-rank adaptation of large language models. ICLR 1:3. doi: 10.48550/arXiv.2106.09685

[B14] Inan H. Upasani K. Chi J. Rungta R. Iyer K. Mao Y. . (2023). Llama guard: LLM-based input-output safeguard for human-ai conversations. arXiv [preprint] arXiv:2312.06674. doi: 10.48550/arXiv.2312.06674

[B15] Kaplan J. McCandlish S. Henighan T. Brown T. B. Chess B. Child R. . (2020). Scaling laws for neural language models. arXiv [preprint] arXiv:2001.08361. doi: 10.48550/arXiv.2001.08361

[B16] Kirk H. R. Gabriel I. Summerfield C. Vidgen B. Hale S. A. (2025). Why human-ai relationships need socioaffective alignment. Humanit. Soc. Sci. Commun. 12, 1–9. doi: 10.1057/s41599-025-04532-5

[B17] Lai T. Shi Y. Du Z. Wu J. Fu K. Dou Y. . (2023). Psy-LLM: Scaling up global mental health psychological services with ai-based large language models. arXiv [preprint] arXiv:2307.11991. doi: 10.48550/arXiv.2307.11991

[B18] Lee J. Yoon W. Kim S. Kim D. So C. H. Kang J. (2020). Biobert: a pre-trained biomedical language representation model for biomedical text mining. Bioinformatics 36, 1234–1240. doi: 10.1093/bioinformatics/btz682 31501885 PMC7703786

[B19] Li Z. Feng W. Guizani M. Yu H. (2024). TPI-LLM: serving 70b-scale LLMs efficiently on low-resource edge devices. arXiv [preprint] arXiv:2410.00531. doi: 10.1109/TSC.2025.3596892

[B20] Liu N. F. Lin K. Hewitt J. Paranjape A. Bevilacqua M. Petroni F. . (2023). Lost in the middle: how language models use long contexts. arXiv [preprint] arXiv:2307.03172. doi: 10.1162/tacl_a_00638

[B21] Liu S.-Y. Wang C.-Y. Yin H. Molchanov P. Wang Y.-C. F. Cheng K.-T. . (2024). “Dora: Weight-decomposed low-rank adaptation,” in Forty-first International Conference on Machine Learning.?

[B22] Machlovi N. Saleki M. Ababio I. Amin R. (2025). Towards safer ai moderation: evaluating LLM moderators through a unified benchmark dataset and advocating a human-first approach. arXiv [preprint] arXiv:2508.07063. doi: 10.48550/arXiv.2508.07063

[B23] Mauriello M. L. Lincoln T. Hon G. Simon D. Jurafsky D. Paredes P. (2021). “Sad: a stress annotated dataset for recognizing everyday stressors in sms-like conversational systems,” in Extended Abstracts of the 2021 CHI Conference on Human Factors in Computing Systems, 1–7.

[B24] Min S. Lyu X. Holtzman A. Artetxe M. Lewis M. Hajishirzi H. . (2022). Rethinking the role of demonstrations: what makes in-context learning work? arXiv [preprint] arXiv:2202.12837. doi: 10.18653/v1/2022.emnlp-main.759

[B25] Miner A. S. Milstein A. Hancock J. T. (2017). Talking to machines about personal mental health problems. JAMA 318, 1217–1218. doi: 10.1001/jama.2017.14151 28973225

[B26] Mitsea E. Drigas A. Skianis C. (2023). Digitally assisted mindfulness in training self-regulation skills for sustainable mental health: a systematic review. Behav. Sci. 13:1008. doi: 10.3390/bs13121008 38131865 PMC10740653

[B27] OpenA I. (2023). Gpt-3.5 Technical Overview.

[B28] Ouyang L. Wu J. Jiang X. Almeida D. Wainwright C. Mishkin P. . (2022). Training language models to follow instructions with human feedback. arXiv [preprint] arXiv:2203.02155. doi: 10.48550/arXiv.2203.02155

[B29] Picard R. Cosier G. (1997). Affective intelligence–the missing link? BT Technol. J. 15, 151–162. doi: 10.1023/A:1018643815520

[B30] Picard R. W. (2000). Affective Computing. Cambridge, MA: MIT Press.

[B31] Pinzuti E. Tüscher O. Castro A. F. (2025). Visually grounded emotion regulation via diffusion models and user-driven reappraisal. arXiv [preprint] arXiv:2507.10861. 10.3389/frai.2026.1691445 PMC12996161 41858847

[B32] Pirina I. Çöltekin Ç. (2018). “Identifying depression on reddit: The effect of training data,” in Proceedings of the 2018 EMNLP Workshop SMM4H: the 3rd Social Media Mining for Health Applications Workshop & *Shared Task*, 9–12.

[B33] Reimers N. Gurevych I. (2019). Sentence-bert: Sentence embeddings using siamese bert-networks. arXiv [preprint] arXiv:1908.10084. doi: 10.18653/v1/D19-1410

[B34] Sanh V. Debut L. Chaumond J. Wolf T. (2019). Distilbert, a distilled version of bert: smaller, faster, cheaper and lighter. arXiv [preprint] arXiv:1910.01108. doi: 10.48550/arXiv.1910.01108

[B35] Sathvik M. Garg M. (2023). Multiwd: Multiple wellness dimensions in social media posts. TechRxiv. doi: 10.36227/techrxiv.22816586 PMC10923126 38191011

[B36] Singhapoo K. Inthanil A. Pillai A. (2025). “Fine-tuning ai models with limited resources,” in 2025 11th International Conference on Engineering, Applied Sciences, and Technology (ICEAST) (Phuket: IEEE), 148–151.

[B37] Taori R. Gulcehre C. Dubois Y. Chang X. Srivastava S. Mirchandani A. . (2023). Alpaca: A Strong, Replicable Instruction-Following Model. Available online at: https://github.com/tatsu-lab/stanford_alpaca

[B38] Tosato T. Helbling S. Mantilla-Ramos Y.-J. Hegazy M. Tosato A. Lemay D. J. . (2025). Persistent instability in LLM's personality measurements: effects of scale, reasoning, and conversation history. arXiv [preprint] arXiv:2508.04826.

[B39] Touvron H. Lavril T. Izacard G. Martinet X. Lachaux M.-A. Lacroix T. . (2023). Llama: Open and efficient foundation language models. arXiv [preprint] arXiv:2302.13971. doi: 10.48550/arXiv.2302.13971

[B40] Turcan E. McKeown K. (2019). Dreaddit: A reddit dataset for stress analysis in social media. arXiv [preprint] arXiv:1911.00133. doi: 10.18653/v1/D19-6213

[B41] Vidgen B. Scherrer N. Kirk H. R. Qian R. Kannappan A. Hale S. A. . (2023). Simplesafetytests: a test suite for identifying critical safety risks in large language models. arXiv [preprint] arXiv:2311.08370. doi: 10.48550/arXiv.2311.08370

[B42] von Platen P. (2023). Transformers Reinforcement Learning (TRL). Available online at: https://github.com/huggingface/trl

[B43] Yang A. Yang B. Hui B. Zheng B. Yu B. Zhou C. . (2024). Qwen2 technical report. arXiv [preprint] arXiv:2407.10671. doi: 10.48550/arXiv.2407.10671

[B44] Yang K. Zhang T. Kuang Z. Xie Q. Huang J. Ananiadou S. (2024). “Mentallama: interpretable mental health analysis on social media with large language models,” in Proceedings of the ACM Web Conference 2024, 4489–4500.

[B45] Yao Y. Duan J. Xu K. Cai Y. Sun Z. Zhang Y. (2024). A survey on large language model (LLM) security and privacy: The good, the bad, and the ugly. High-Confidence Comp. 4:100211. doi: 10.1016/j.hcc.2024.100211

[B46] Yu Z. Wang Z. Li Y. Gao R. Zhou X. Bommu S. R. . (2024). “Edge-LLM: Enabling efficient large language model adaptation on edge devices via unified compression and adaptive layer voting,” in Proceedings of the 61st ACM/IEEE Design Automation Conference, 1–6.

[B47] Zhang B. Liu Z. Cherry C. Firat O. (2024). When scaling meets LLM finetuning: the effect of data, model and finetuning method. arXiv [preprint] arXiv:2402.17193. doi: 10.48550/arXiv.2402.17193

[B48] Zhang Q. Chen M. Bukharin A. Karampatziakis N. He P. Cheng Y. . (2023). AdaLoRA: adaptive budget allocation for parameter-efficient fine-tuning. arXiv [preprint] arXiv:2303.10512. doi: 10.48550/arXiv.2303.10512

